# RETRACTION: The Role of Orthobiologics in Chronic Wound Healing

**DOI:** 10.1111/iwj.70744

**Published:** 2025-08-06

## Abstract

**RETRACTION:** DominguesR. Barnabé , von RautenfeldM. , KavalcoC. M. , CaliariC. , DellagiustinaC. , , Furtado da FonsecaL , CostaF. Ramos , da Cruz Silva ReisA. , SantosG. Silva , AzziniG. , de FariaA. Pinto Lemos , SantosN. , PiresL. , HuberS. Cares , MahmoodA. , DalloI. , EvertsP. , and LanaJ. Fábio , “The Role of Orthobiologics in Chronic Wound Healing,” International Wound Journal 21, no. 4 (2024): e14854, 10.1111/iwj.14854.38619232 PMC11017856

The above article, published online on 15 April 2024 in Wiley Online Library (http://onlinelibrary.wiley.com/), has been retracted by agreement between the journal Editor in Chief, Professor Keith Harding; and John Wiley & Sons, Ltd. An investigation by the publisher found that the article contained significant textual overlap between this article and other articles by different authors [1–3]. Additional textual overlap was also detected in multiple other articles [4–7]. While each of the reference articles noted in this retraction was included as a reference in the published article, the overlap extended to entire passages that were copied verbatim and not included in quotation marks. The authors responded to an inquiry by the publisher and stated that some technical language and common terms for physiological mechanisms and biochemical actions of orthobiologics may have contributed to the findings of significant textual overlap.

A review of the textual overlap by the publisher found that it constituted significant plagiarism which could not be amended through correction. As such, all parties have agreed that the article must be retracted. The authors did not agree with the retraction. The authors disagree with the retraction.

**References:**

[1] 
FrykbergR. G.
 and 
BanksJ.
, “Challenges in the Treatment of Chronic Wounds,” Advances in Wound Care
4, no. 9 (2015): 560–582, 10.1089/wound.2015.0635.26339534
PMC4528992

[2] 
JoH.


BritoS.


KwakB. M.


ParkS.


LeeM.‐G.


BinB.‐H.

Applications of Mesenchymal Stem Cells in Skin Regeneration and Rejuvenation
International Journal of Molecular Sciences
22 no. 5
2021
2410
10.3390/ijms22052410
33673711
PMC7957487

[3] 
EvertsP.


OnishiK.


JayaramP.


LanaJ. F.


MautnerK.

Platelet‐Rich Plasma: New Performance Understandings and Therapeutic Considerations in 2020
International Journal of Molecular Sciences
21
20
2020
7794
10.3390/ijms21207794
33096812
PMC7589810

[4] 
EvertsP.


SadeghiP.


SmithD. R.

Basic Science of Autologous Orthobiologics: Part 1. Platelet‐Rich Plasma
Physical Medicine and Rehabilitation Clinics of North America
34
1
2023
1
23
10.1016/j.pmr.2022.08.003
36410877

[5] 
PopescuV.
, 
CauniV.
, 
PetrutescuM. S.
, 
RustinM. M.
, 
BocaiR.
, 
TurculetC. R.
, 
DoranH.
, 
PatrascuT.
, 
LazarA. M.
, 
CretoiuD.
, 
VarlasV. N.
, and 
MastalierB.
, “Chronic Wound Management: From Gauze to Homologous Cellular Matrix,” Biomedicines
11, no. 9 (2023): 2457, 10.3390/biomedicines11092457.37760898
PMC10525626

[6] 
LanaJ. F. S. D.
, 
LanaA. V. S. D.
, 
da FonsecaL. F.
, 
CoelhoM. A.
, 
MarquesG. G.
1, 
MosanerT.
, 
RibeiroL. L.
, 
AzziniG. O. M.
, 
SantosG. S.
, 
FonsecaE.
, and 
de AndradeM. A. P.
, “Stromal Vascular Fraction for Knee Osteoarthritis – An Update,” Journal of Stemcells & Regenerative Medicine
18, no. 1 (2022): 11–20, 10.46582/jsrm.1801003.PMC937935736003656

[7] 
Martinez‐ZapataM. J.


Martí‐CarvajalA. J


SolàI.


ExpósitoJ. A.


BolíbarI.


RodríguezL.


GarciaJ.


ZarorC.

Autologous Platelet‐Rich Plasma for Treating Chronic Wounds
Cochrane Database of Systematic Reviews
2016
2016
CD006899
10.1002/14651858.CD006899.pub3
5.27223580
PMC9308064

**RETRACTION:** R. Barnabé Domingues , M. von Rautenfeld , C. M. Kavalco , C. Caliari , C. Dellagiustina , L, Furtado da Fonseca , F. Ramos Costa , A. da Cruz Silva Reis , G. Silva Santos , G. Azzini , A. Pinto Lemos de Faria , N. Santos , L. Pires , S. Cares Huber , A. Mahmood , I. Dallo , P. Everts , and J. Fábio Lana , “The Role of Orthobiologics in Chronic Wound Healing,” International Wound Journal 21, no. 4 (2024): e14854, 10.1111/iwj.14854.38619232 PMC11017856

The above article, published online on 15 April 2024 in Wiley Online Library (http://onlinelibrary.wiley.com/), has been retracted by agreement between the journal Editor in Chief, Professor Keith Harding; and John Wiley & Sons, Ltd. An investigation by the publisher found that the article contained significant textual overlap between this article and other articles by different authors [1–3]. Additional textual overlap was also detected in multiple other articles [4–7]. While each of the reference articles noted in this retraction was included as a reference in the published article, the overlap extended to entire passages that were copied verbatim and not included in quotation marks. The authors responded to an inquiry by the publisher and stated that some technical language and common terms for physiological mechanisms and biochemical actions of orthobiologics may have contributed to the findings of significant textual overlap.

A review of the textual overlap by the publisher found that it constituted significant plagiarism which could not be amended through correction. As such, all parties have agreed that the article must be retracted. The authors did not agree with the retraction. The authors disagree with the retraction.
